# Identifying Schizophrenia Using Structural MRI With a Deep Learning Algorithm

**DOI:** 10.3389/fpsyt.2020.00016

**Published:** 2020-02-03

**Authors:** Jihoon Oh, Baek-Lok Oh, Kyong-Uk Lee, Jeong-Ho Chae, Kyongsik Yun

**Affiliations:** ^1^ Department of Psychiatry, Seoul St. Mary’s Hospital, College of Medicine, The Catholic University of Korea, Seoul, South Korea; ^2^ Department of Ophthalmology, Seoul National University Hospital, Seoul, South Korea; ^3^ Department of Psychiatry, Uijeongbu St. Mary’s Hospital, College of Medicine, The Catholic University of Korea, Seoul, South Korea; ^4^ Computation and Neural Systems, California Institute of Technology, Pasadena, CA, United States; ^5^ Bio-Inspired Technologies and Systems, Jet Propulsion Laboratory, California Institute of Technology, Pasadena, CA, United States

**Keywords:** schizophrenia, deep learning, MRI, classification, structural abnormalities

## Abstract

**Objective:**

Although distinctive structural abnormalities occur in patients with schizophrenia, detecting schizophrenia with magnetic resonance imaging (MRI) remains challenging. This study aimed to detect schizophrenia in structural MRI data sets using a trained deep learning algorithm.

**Method:**

Five public MRI data sets (BrainGluSchi, COBRE, MCICShare, NMorphCH, and NUSDAST) from schizophrenia patients and normal subjects, for a total of 873 structural MRI data sets, were used to train a deep convolutional neural network.

**Results:**

The deep learning algorithm trained with structural MR images detected schizophrenia in randomly selected images with reliable performance (area under the receiver operating characteristic curve [AUC] of 0.96). The algorithm could also identify MR images from schizophrenia patients in a previously unencountered data set with an AUC of 0.71 to 0.90. The deep learning algorithm’s classification performance degraded to an AUC of 0.71 when a new data set with younger patients and a shorter duration of illness than the training data sets was presented. The brain region contributing the most to the performance of the algorithm was the right temporal area, followed by the right parietal area. Semitrained clinical specialists hardly discriminated schizophrenia patients from healthy controls (AUC: 0.61) in the set of 100 randomly selected brain images.

**Conclusions:**

The deep learning algorithm showed good performance in detecting schizophrenia and identified relevant structural features from structural brain MRI data; it had an acceptable classification performance in a separate group of patients at an earlier stage of the disease. Deep learning can be used to delineate the structural characteristics of schizophrenia and to provide supplementary diagnostic information in clinical settings.

## Introduction

Structural brain alterations in schizophrenia have been thoroughly investigated with the development of neuroimaging methods (1–3). Although there remain some controversies regarding the use of antipsychotics and the duration of illness, a number of studies have found overall gray matter loss (2), decreased volume of the bilateral medial temporal areas (3) and a left superior temporal region deficit (1) in brains with schizophrenia. As these structural abnormalities are thought to be linked to the positive symptoms of schizophrenia (4, 5), it has been suggested that the neuropathology and etiology of schizophrenia might be related to alterations in brain structure (6).

Although studies on volumetric magnetic resonance imaging (MRI) analysis in schizophrenia have shown relatively consistent results over several decades (7), diagnosing schizophrenia based on these findings is still challenging and has little clinical utility. One possible reason is that the predictive value of biological features of schizophrenia weakens in real-world patients who have symptoms superficially resembling those of other psychiatric illnesses (8). Multiple internal phenotypes of schizophrenia, such as electrophysiological properties (P50, P300, and mismatch negativity), achieved a high diagnostic accuracy of approximately 80%, but these features were studied in relation to genetic analysis rather than clinical application (9). Another reason is that certain cortical features found in schizophrenia are shared with other neurodegenerative diseases; thus, the patient’s clinical history of psychiatric problems is needed to discriminate these mental illnesses (10).

Recent machine learning methods continue to address these issues. As deep learning algorithms have achieved superior performance in visual image recognition (11), their clinical significance has increased in certain diagnostic tasks, such as detecting pulmonary nodules on chest CT scans (12) and diagnosing diabetic retinopathy from retinal fundus photographs (13). Similar studies have been conducted in schizophrenia patients using structural MRI data, and acceptable accuracy rates have been achieved (68.1% to 85.0%) (14–17). A deep belief network achieved a higher accuracy rate than a classical machine learning algorithm in discriminating schizophrenia patients from healthy controls (15). One of the important characteristics of deep learning is that it learns through labeled images and identifies important features without explicitly designated characteristics (11, 18), and it learns representations of input data as the information flow ascends through multiple layers (11). Therefore, in order to infer the cortical features of schizophrenia using deep learning algorithms, it is necessary to examine how such a decision is made and compare those findings with the results of volumetric MRI studies.

In this study, we trained a deep learning algorithm (19) to identify schizophrenia using five multicenter data sets of structural MRI results and assessed the classification performance of the algorithm in a single-center, clinical validation set. Furthermore, we examined which brain regions mainly contributed to the decisional process of the deep learning algorithm.

## Methods

### Data Sets

Publicly available neuroimaging data from schizophrenia patients and normal subjects were obtained from the SchizConnect (https://www.schizconnect.org) database (20). Among 1,392 sets of data from subjects in this database, we used 873 sets of structural MRI information available from 5 multicenter data sets, i.e., BrainGluSchi (21), COBRE (22), MCICShare (23), NMorphCH (24) and NUSDAST (25). These data sets had been acquired to investigate the brain metabolism of patients with schizophrenia (BrainGluSchi) and included both structural and functional images (COBRE and MCICShare). The structural images were obtained from 1998 to 2016, and the scanner field strength varied among data sets (1.5 T and 3 T, **Table 1**). All raw images were evaluated by the authors of the present study, and images not applicable for training the deep learning algorithm (e.g., those with excessive motion or noise or an image error) were excluded (**Table 1**). The study protocol was approved by the Institutional Review Board of Seoul St. Mary’s Hospital (KC18ZESI0615).

**Table 1:** Sample characteristics of 5 public schizophrenia MRI and validation data sets.

Characteristics		BrainGluSchi	COBRE	MCICShare	NMorph	NUSDAST	Validation data set
No. of images		175	184	204	90	220	60
No. of normal images		89	94	95	44	102	30
No. of schizophrenia images		86	90	109	46	118	30
Patient demographics							
	Age, mean (SD), years	36.7 (14.2)	38.3 (12.6)	33.9 (11.6)	31.9 (7.8)	32.9 (12.0)	31.9 (7.2)
	Age, range (min/max)	16/65	18/66	18/61	19/46	14/66	22/50
	Female, No./total (%)	36 / 175 (20.6)	45 / 184 (24.5)	56 / 190 (29.5)	36 / 90 (40.0)	84 / 218 (38.5)	35 / 60 (58.3)
Image quality							
	Acquisition year	2010 to 2013	2009 to 2013	2004 to 2006	2008 to 2013	1998 to 2006	2014 to 2016
	Scanner field strength	3 T	3 T	1.5 T/3 T	3 T	1.5 T	1.5 T
	No. with excessive motion	–	–	–	2	–	–
	No. with excessive noise	–	1	1	–	1	–
	No. with image errors	–	–	–	–	2	–
Psychiatric diagnosis							
	Schizophrenia (broad)^a^	86	–	95	–	–	–
	Schizophrenia (strict)	–	79	–	44	117	30
	Schizoaffective disorder	–	11	–	2	–	–
Disease characteristics^b^							
	Duration of illness (SD), year	–	N/A	10.67 (10.3)	N/A	–	4.89 (3.47)
	Duration of treatment (SD), month	–		–		–	14.7 (18.8)
	Antipsychotic use (%)	93.3		–		–	100.0
	PANSS (SD)^c^	–		–		–	54.9 (28.4)
	SAPS (SD)^d^	–		4.96 (2.77)		11.1 (12.7)	–
	SANS (SD)^e^	–		8.00 (3.91)		9.6 (10.7)	–
	GAF score (SD)^f^	–		–		–	62.8 (12.3)

a Includes both schizophrenia and schizoaffective disorder.

b Disease characteristics of public data sets represent whole patients of each study.

c Positive and Negative Syndrome Scale.

d Scale for the Assessment of Positive Symptoms.

e Scale for the Assessment of Negative Symptoms.

f Global Assessment of Functioning.

**Figure 1:**
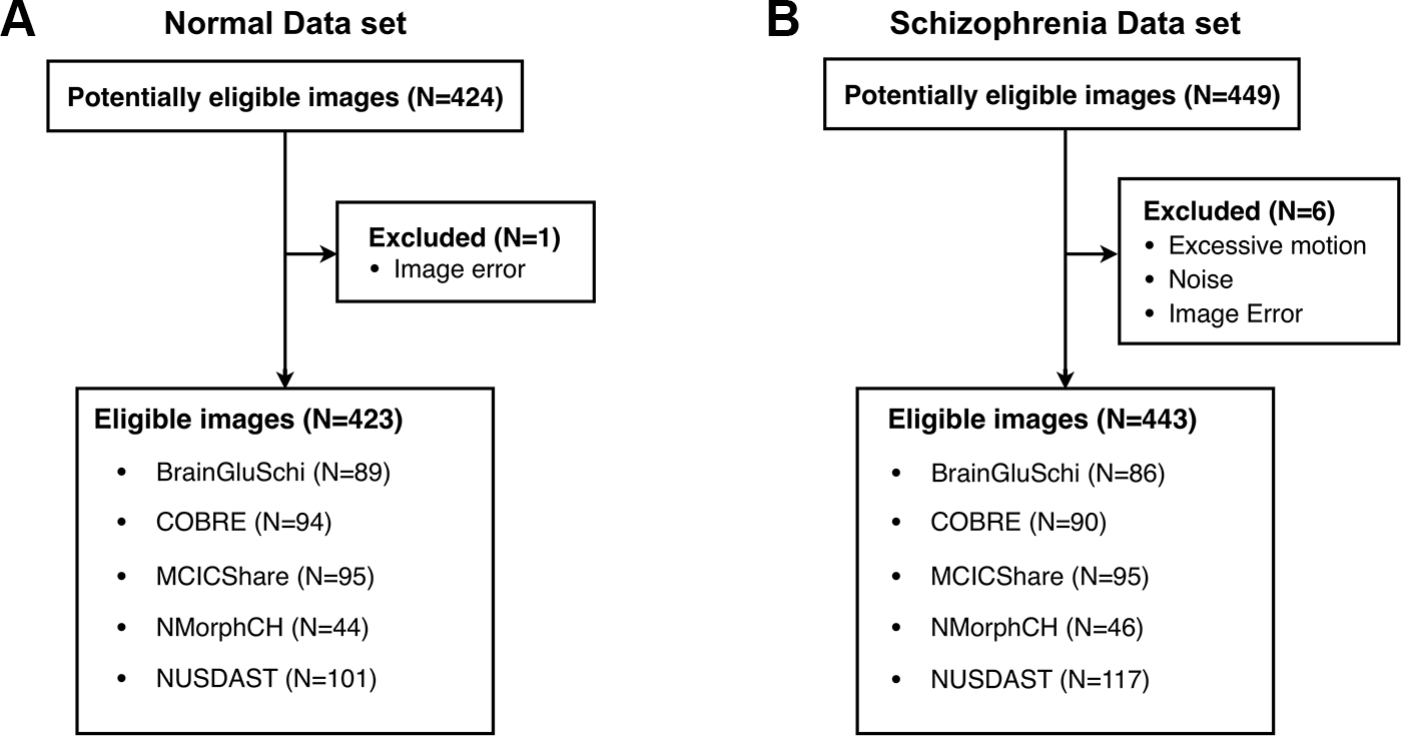
Five public MRI data sets for the detection of schizophrenia through a deep learning algorithm. **(A)** Normal data sets consisted of structural MR images obtained from healthy control subjects. **(B)** Schizophrenia data sets consisted of structural MR images obtained from schizophrenia and schizoaffective disorder patients.

Among a total of 873 raw images (449 of patients with schizophrenia, 424 of normal subjects), seven images with excessive motion and noise were excluded (**Figure 1**). Thus, 866 eligible images (443 of patients with schizophrenia, 423 of normal subjects) were used to train the deep learning model (**Table 1**). All MRI data were acquired by high-resolution T1-weighted structural magnetization-prepared rapid gradient-echo (MPRAGE) scans, but the scanning parameters varied across data sets (**Supplementary Table 1**).

Among 449 data sets from schizophrenia patients in the training data set, 181 were categorized as the “schizophrenia (broad)” group, and 240 were categorized as the “schizophrenia (strict)” group. Schizophrenia (broad) refers to the diagnosis of both schizophrenia and schizoaffective disorder (20), although this distinction was not included in the clinical diagnosis. We included the data of the schizophrenia (broad) group in the analysis because these two diseases may share similar characteristics and disease courses (26) and have usually been included together in imaging studies (27).

The validation data set was acquired in a single center in South Korea and consisted of data from 30 patients with schizophrenia and 30 healthy controls (**Table 1**). As this data set had detailed information on each subject, we could evaluate the severity of disease, duration of treatment, and use of antipsychotics. The patients in this data set were “mildly ill” and had “some mild symptoms” in their lives, as assessed by the Positive and Negative Symptom Scale (PANSS) (28) and Global Assessment of Functioning (GAF) (29), respectively. This data set also had some demographic differences from the 5 multicenter data sets from the SchizConnect database; in particular, the validation data set included relatively younger subjects (mean [SD]: SchizConnect = 35.3 [12.6], Uijeongbu St. Mary’s = 31.9 [7.2]) and had a higher proportion of females (ratio of females; SchizConnect = 23.4%, Uijeongbu St. Mary’s = 58.3%). These features of the validation set enabled us to verify whether the trained deep learning model could flexibly cope with a new situation, i.e., a data set with different disease characteristics than the training data sets.

### Image Preprocessing

All Nifti images were manually evaluated by the authors using MRIcron software (http://www.cabiatl.com/mricro/mricron/index.html). The slice number on the z-axis that corresponded to the top of the skull and the x-y coordinates of the midbrain were measured on the coronal view of each image. Then, each slice of the transverse section was converted to a frame, and these frames were combined into a video (**Supplementary Figure 1** and **Supplementary Video 1**) using MATLAB software (MathWorks, Inc., MA). The image intensity of each video was normalized within the data set (i.e., the mean image intensity was equalized between MR images of normal subjects and patients with schizophrenia) to prevent the algorithm from classifying diseases based on the basic properties of images.

We used a series of videos rather than the entire 3D Nifti image as the input for the following reasons. First, we aimed to reproduce the way clinicians actually read brain MR images. Clinicians do not interpret the MR images as a whole but examine the pre-post slices in a serial process. As deep learning essentially imitates the structure of the human cortex and the information processing of the brain (30), we decided that the inputs provided to the deep learning algorithm should be similar to those that humans would actually experience (31).

### Development of The Algorithm

A three-dimensional convolutional neural network (3DCNN) architecture was used for classifying patients with schizophrenia and normal subjects based on the structural MRI data sets (32–34); the original 3DCNN architecture was developed for video classification (https://github.com/kcct-fujimotolab/3DCNN). The input to the 3DCNN was a converted video of a subject’s structural MR images (concatenated slices along the z-axis; **Supplementary Figure 1**). The input dimensions were 256 × 256 × 180. This architecture has four 3D convolutional layers, with max-pooling-based downsampling in each convolutional layer. A previous study using the ADNI data set showed that 3DCNNs with only one convolutional layer outperformed other classifiers in predicting the Alzheimer’s disease status of a patient based on an MRI scan of the brain (35). More recently, four 3DCNNs were used in high-precision segmentation and classification problems, reportedly achieving state-of-the-art performance (36, 37). We applied a rectified linear unit (ReLU) activation function, which is the most commonly used activation function in deep learning models. The function returns 0 if it receives any negative input, but for any positive value *x*, it returns the input value, as follows: f(*x*) = max(0, *x*). This activation function is known to effectively capture the interactions and nonlinearities of data sets (11, 38).

The kernel size was 3 × 3 × 3, and the pooling size was the same. The kernel size was selected to match the Gaussian kernel size used for MRI postprocessing to reduce artifacts (39). We applied the parameters of depth = 15 and color = true settings, so that the first layer was 32 × 32 × 15 × 3. The original input had 11 million parameters (256 × 256 × 180 = 11,796,480), and it was downsampled to 30,720 parameters (32 × 32 × 15 × 3). Thus, there was 384× parameter reduction (11,796,480/30,720=384).

The ReLU activation function and a dropout rate of 0.25 were used for each convolutional layer. At the end of the convolutional layers, one densely connected layer with a dropout rate of 0.5 was attached. The models were trained for 50 epochs with a batch size of 32 (40). The training was stopped as soon as convergence was achieved (epochs = 50, **Supplementary Figure 4**) to avoid unexpected overfitting that could confound the results. Previous studies have shown that stopping the training process early could potentially improve the generalization (41, 42). The learning rate and momentum for stochastic gradient descent (SGD) were set to 0.001 and 0.9, respectively. All trainings and experiments were run on a standard workstation (64 GB RAM, 3.30 GHz Intel Core i7 CPU, NVidia GTX 1080, 8 GB VRAM). Model training ran for ~30 hours. A full model of the deep learning algorithm is presented in **Supplementary Figure 2**, and the algorithms used in this study have been uploaded to a developer community and can be freely downloaded (https://github.com/yunks128/3D-convolutional-neural-networks).

### Evaluating Algorithms Using The Training and Validation Data Sets

**Figure 2:**
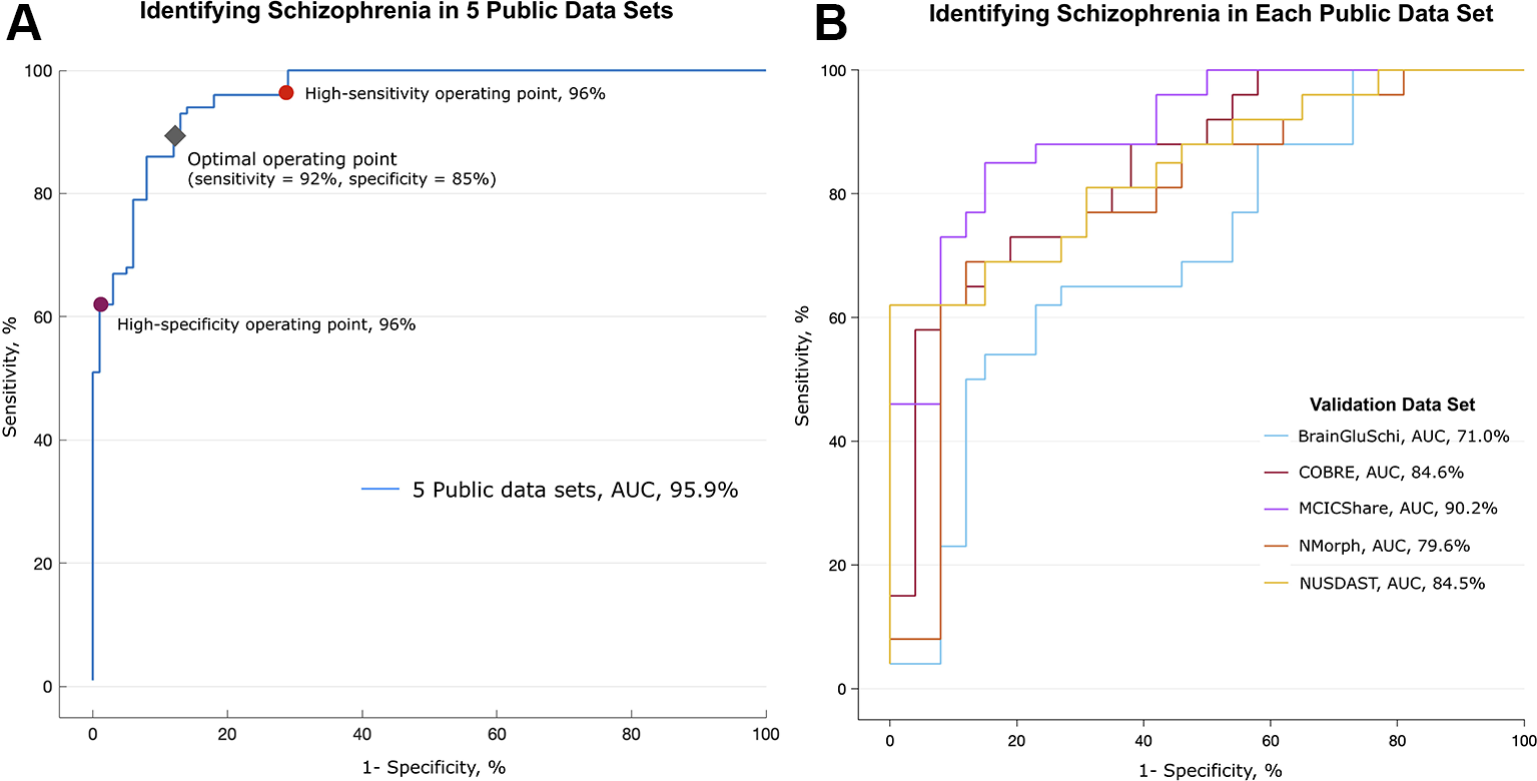
Performance in detecting schizophrenia in five public MRI data sets. Performance in identifying schizophrenia in five publicly available MRI data sets. **(A)** The deep learning algorithm was trained with 693 randomly selected images (80% of all images) and discriminated between patients with schizophrenia and normal subjects in the remaining 173 MR images. This process was repeated 10 times (10-fold cross-validation). The area under the receiver operating characteristic (ROC) curve (AUC) was 0.959. The red and purple circles on the graph represent optimal operating points; the sensitivity was 96% and the specificity was 96% at these points, respectively. The gray diamond represents the optimal operating point, which had 92% sensitivity and 85% specificity. **(B)** Validation of algorithm performance across the data sets. The deep learning algorithm was trained with MR images from four of five data sets, and the remaining one data set was used as a validation set. The algorithm trained without the MCICShare data set showed the highest performance (red line, AUC of 0.902), and the algorithm trained without the BrainGluSchi data set showed the lowest performance (blue line, AUC of 0.710).

To avoid the overfitting problem (43), we applied 10-fold cross-validation in training the deep learning algorithm (44). The original data set was randomly partitioned into 10 equally sized data subsets, and a single data subset was used as the validation set for testing the model trained with the other data subsets (**Figure 2A**). We also applied cross-validation to each of the five data sets individually; one of the five data sets was designated as a test set, and the remaining four were used for training. In this validation, the deep learning model was trained with four of five data sets, and a remaining data set was used as a test set. For example, the deep learning model trained with the COBRE, MCICShare, NUSDAST, and NMorph data sets was assessed for the ability to identify schizophrenia in the BrainGluSchi data set. This method enabled us to evaluate whether the trained deep learning algorithm could classify structural images obtained from schizophrenia patients with different scanning parameters and scanner field strengths (**Figure 2B**).

To determine whether the trained deep learning algorithm could distinguish patients with schizophrenia from healthy controls in real-world MRI data sets, we used a new data set obtained by Uijeongbu St. Mary’s Hospital in South Korea. This data set consisted of structural MRI data from 30 schizophrenia patients and 30 healthy controls (**Table 1**).

### Regional Analysis

**Figure 3:**
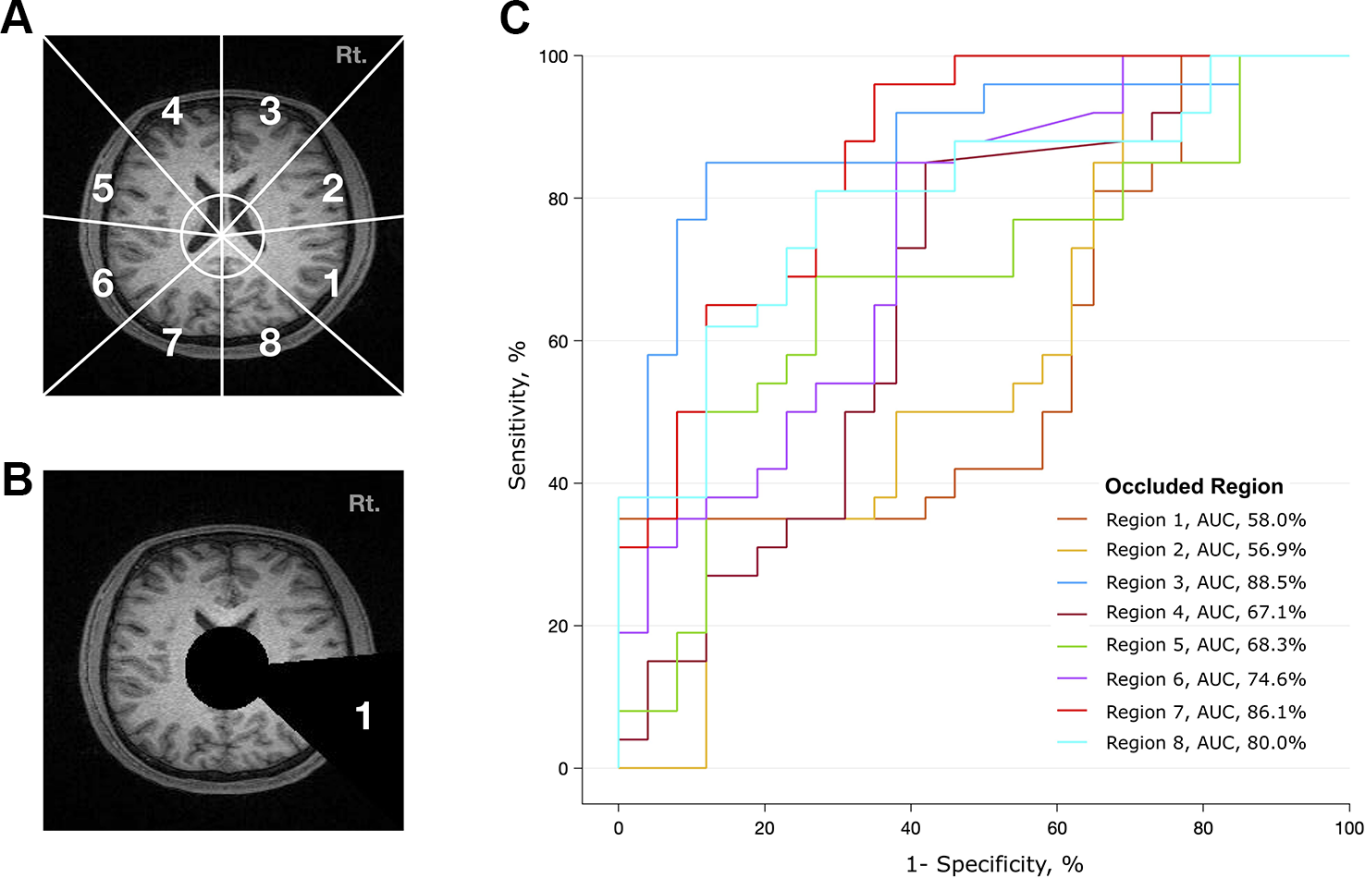
Analysis of contributing brain regions for detecting schizophrenia. Contribution of each brain region in identifying MR images from patients with schizophrenia. Each MR transverse slice was divided into eight regions, and one of these regions was occluded with a black triangle. Thus, no information was provided from this portion of the brain. The deep learning algorithm was trained with these handicapped inputs and subsequently used to classify MR images. **(A)** Schematic diagram of eight arbitrarily determined brain regions. The center of the circle corresponds to the center of the midbrain, and the endpoint of each line corresponds to a vertex and midpoint of the image. **(B)** A sample slice that was used as an input to the algorithm. Areas corresponding to ventricles and region 1 are covered. **(C)** Performance of the deep learning algorithm. Region 1 mostly contributed to identifying schizophrenia, as the performance dropped to an AUC of 0.58 when the information from region 1 was not provided.

To determine which brain regions contributed most to the classification, we divided the transverse section of the MR image into eight regions (**Figure 3A**). Based on the x-y coordinates of the midbrain, a black circle with a radius of 30 pixels was drawn in each frame. Then, a black triangle with end points at the center of the circle and at a vertex and midpoint of the image was drawn in every frame. We made a total of eight different videos in which the triangle occluded eight different regions (**Figure 3B** and **Supplementary Video 2**). Transverse brain sections with one of the eight areas occluded were used to train the deep learning algorithm. Then, we evaluated how the performance of the deep learning algorithm changed in distinguishing patients with schizophrenia from healthy controls. If the area under the receiver operating characteristic curve (AUC) and accuracy rate significantly dropped, we could infer that the structural MRI information in that area mainly contributed to identifying schizophrenia. This method is more arbitrary than using the parcellation of cortical areas from 3D Nifti images (45), but it is advantageous in that it reduces the processing time and resources needed to analyze a large amount of MRI data.

### Logistic Regression Algorithm For Validation

Although we normalized the mean intensity of all MR images, one may argue that simple MRI intensity differences between the schizophrenia and normal groups may provide significant classification power. To test whether information on mean intensity could be used to determine whether a given subject has schizophrenia, we independently applied the logistic regression classifier. The method estimates the log odds of an event that can be mathematically expressed as a multiple linear regression function. Let the predictor X_1_ be the mean image intensity, and let the binary response variable Y be the output of either schizophrenia or normal, where the probability of Y is denoted as p = P(Y=1). The log odds, L, can be written as follows (where B_0_ and B_1_ are parameters of the model):

$$L=\log\left(\frac{p}{1-p}\right)=B_{0}+B_{1}X_{1}$$

### Clinician Rating Experiments

To investigate whether clinicians could identify the imaging characteristics of schizophrenia, we presented one hundred randomly selected videos (50 from schizophrenia patients and 50 from normal subjects) to seven clinicians (five psychiatrists and two radiologists). The clinicians were required to rate the likelihood that the presented video was from a schizophrenia patient as a number from 0 to 100. Before the rating, they were told the main characteristics of the brain of patients with schizophrenia but were naïve to diagnosing schizophrenia based on brain MRI data. To determine whether there is a learning effect in the classification performance of humans, the same psychiatrist performed 3 consecutive experiments. After each session, he/she was provided with the correct answers. Each session consisted of 100 randomly selected videos, which were completely different in each session.

## Results

In the training data set, the deep learning model achieved high performance in classifying the structural MRI data of schizophrenia vs. normal subjects (AUC of 0.96, **Figure 2A**). The overall accuracy rate was 97%, meaning that among 866 images, 840 images were classified correctly. The probability of randomly selected images being classified as schizophrenia by chance was 51.2% (443 schizophrenia and 423 normal images). The sensitivity of the algorithm at the high-sensitivity operating point was 96%, and the specificity at the high-specificity operating point was 96%. The sensitivity and specificity at the optimal operating point was 92% and 85%, respectively (gray diamond in **Figure 2A**). The mean image intensity (range, 0 to 255) across all images in the schizophrenia group was 52.52 (SD = 23.68), and that in the normal group was 50.40 (SD = 22.57). The logistic regression machine learning algorithm failed to classify schizophrenia and normal subjects (accuracy rate = 51.2%, chance level = 51.1%) in these data sets; thus, image quality and intensity did not affect the classification performance.

To test the performance of the algorithm across each training data set, we further evaluated the classification performance using a separate data set as the test set (**Figure 2B**). Deep learning achieved the highest classification performance when the MCICShare data set was presented as a new input (AUC of 0.90) and showed the lowest performance in classifying the BrainGluSchi data set (AUC of 0.71). These results suggest that the data sets contributed unequally to classifying the characteristics of schizophrenia; the BrainGluSchi data set might contain crucial information for distinguishing patients with schizophrenia from normal subjects.

**Figure 4:**
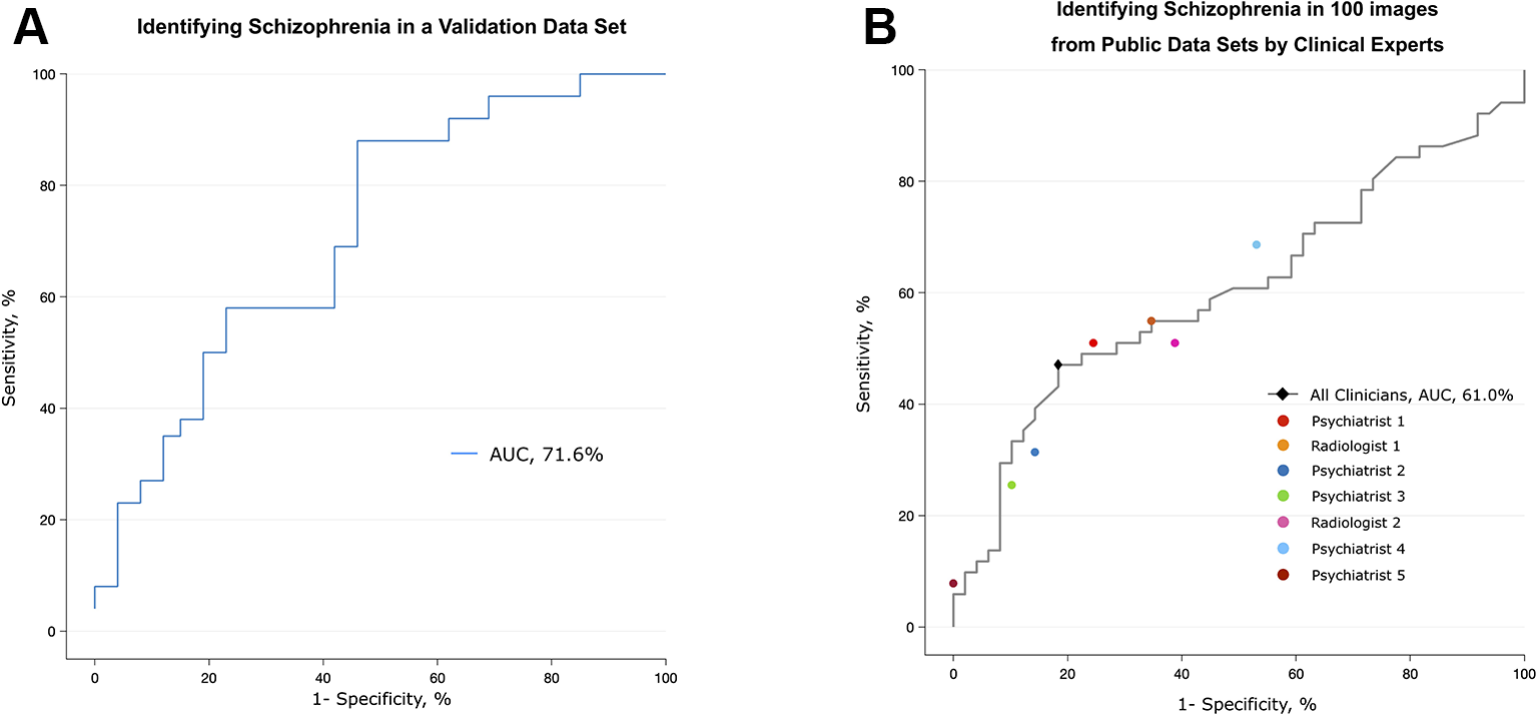
Validation of the algorithm using a different data set and the performance of clinical specialists. **(A)** The algorithm trained with five public data sets discriminated scans from patients with schizophrenia and normal subjects in the validation data set, which consisted of patients who were younger and at an earlier stage of the disease. **(B)** Classification performance of clinical specialists who had been semitrained regarding the structural characteristics of the brain in schizophrenia. The black diamond highlights the optimal operating point of all humans (sensitivity = 81.6%, specificity = 47.1%), and each colored circle shows the optimal operating point of each individual.

The performance of the deep learning algorithm that identified patients with schizophrenia was somewhat lowered in a completely new data set (Uijeongbu St. Mary’s). When the deep learning algorithm had a new input from the validation set consisting of 60 structural images that had slightly different disease characteristics relative to the training sets, its predictive AUC value dropped from 0.95 to 0.72 (**Figure 4A**). The accuracy rate of the deep learning algorithm in the validation data set was 70.0%, compared to a 50% chance level. This finding shows that the predictive power of the deep learning algorithm significantly decreased when it encountered MRI information completely different from the data used for training.

To investigate whether the spatial information of an individual MRI data set affects the classification ability of the deep learning algorithm, we further analyzed the regional data. **Figure 3** summarizes the process and the results of this analysis. Among the eight occluded areas, when the area marked by 1 (roughly corresponding to the right temporal region) was colored black with zero image intensity, the AUC dropped from 0.96 to 0.57, which was the largest change. In contrast, when the area marked by 3 was occluded, the change in the AUC was smaller (0.96 to 0.89); this area contained the right frontal region.

Although clinicians rarely diagnose schizophrenia based on structural MRI alone, we tested whether experts in related fields can distinguish between MR images from patients with schizophrenia and normal subjects. Seven clinical specialists, five psychiatrists and two radiologists, were briefly told the known findings of brain abnormalities in schizophrenia and were given 100 randomly selected videos (50 of patients with schizophrenia, 50 of normal subjects) that were identical to those used in training the deep learning algorithm. The overall accuracy rate of the seven specialists was 62% (AUC of 0.61), which was barely over the chance level (50%) (**Figure 4B** and **Supplementary Table 2**). The sensitivity and specificity at the optimal operating point was 81.6% and 47.1%, respectively. The test for a learning effect in the psychiatrist showed that there was no improvement in the AUC as the sessions proceeded (**Supplementary Figure 3**).

## Discussion

Our results imply that a deep learning algorithm trained with large structural MRI data sets could discriminate patients with schizophrenia from healthy participants. Without any explicit instructions or lesion-related information, deep neural networks can learn and find relevant brain regions that mainly contribute to the identification of scans from patients with schizophrenia. Interestingly, the brain regions that most affected the deep learning algorithm (the right temporal and right temporoparietal areas) corresponded to previous findings of voxel-based analyses.

Although our results are still incomplete regarding application for the clinical diagnosis of schizophrenia, they have several advantages. The deep neural network trained with structural MRI data sets achieved high sensitivity and specificity (96% and 96%, respectively), higher than those obtained in previous studies that used other machine learning algorithms (accuracy rate of 85.0%) (14) and similar deep belief networks (AUC of 0.79) (15). This improvement in classification performance might have been related to the number of images in our study being larger than that in the previous study (143 patients in the study by Pinaya et al. (15) vs. 443 patients in this study), as the performance of deep learning improves when more data become available (46).

The classification performance was relatively acceptable in the five multicenter data sets provided by the SchizConnect database, but the performance degraded in the data set from the single center acquired in South Korea (accuracy rate of 70.0% and AUC of 0.72). Because the trained deep learning algorithm had consistently shown high performance even in data sets with different scanning parameters and scanner field strengths (e.g., identifying schizophrenia in the COBRE data set with algorithms trained on the other 4 multicenter data sets, **Figure 2B**), these results do not ensure that the predictive ability of trained deep learning algorithms is limited. Rather, the change in performance might be related to the different disease characteristics in the patients included in the data sets. In the MCICShare data set, the illness duration of the patients with schizophrenia was 10.67 (SD = 10.03) years (23), but patients in the validation data set from Uijeongbu St. Mary’s hospital had an average illness duration of 4.89 (3.47) years. The age range of patients in the validation set was smaller (22 to 50) than that in the training data sets (16 to 66) (**Table 1**). Thus, it can be inferred that the schizophrenia patients in the validation data set were younger and had less advanced disease than those in the training set. As there are progressive morphometric changes in the brain of a schizophrenia patient over time (47), structural abnormalities in the validation data set could have been somewhat smaller than those in the training data sets. These substantial differences in participant characteristics between the training and validation data sets might degrade the classification ability of the deep learning algorithm.

Regional analysis showed that different brain regions contributed unequally to identifying schizophrenia (**Figure 3**). The area that includes the right temporal region (marked as #1) contributed the most to discriminating between scans from patients with schizophrenia and normal subjects, followed by the right temporoparietal (marked as #2) and left frontal (marked as #4) regions. Information from the left occipital (marked as #7) and right frontal (marked as #3) areas made small contributions, as the AUC was largely preserved (> 0.86) when these regions were treated as “null.” These results correspond to the findings of voxel-based meta-analyses of brain images from subjects with schizophrenia (2, 3, 48). Shenton et al. found definite brain abnormalities in schizophrenia patients, especially in the medial temporal lobe (74% of studies reviewed), and 100% of reviewed studies reported abnormalities in the superior temporal gyrus (48). However, there was moderate evidence of abnormalities in the frontal and parietal lobes, as approximately 60% of the reviewed studies reported findings to that effect (48).

In this study, the deep learning algorithm was informed of only the label of each input video (“schizophrenia” or “normal”), and no other explicit instructions were given. Thus, the deep learning algorithm identified certain brain characteristics of schizophrenia on its own during the training and used this information to classify brain MR images. Although we used qualitative methods rather than precise cortical parcellation to divide brain regions (49), these results suggest that a deep learning algorithm could be used to identify certain brain features of schizophrenia, complementing the findings of previous studies.

Identifying schizophrenia using structural MR images is uncommon in clinical settings. The diagnosis of schizophrenia mainly depends on the psychiatrist’s detailed interview with patients and his/her family and the use of systematic diagnostic tools (29). The relatively low performance of the clinicians in this study may have been because they were not at all familiar with identifying the disease through MR images. Although clinical specialists were made aware of several cortical features of the brain in schizophrenia, they were not equally skilled competitors with the deep learning algorithm. The format of the videos, in which pre-post slices could not be freely investigated (which is possible in the PACS framework), could have also contributed to the difficulty experienced by clinicians in identifying certain features of schizophrenia. Thus, the poor classification rate of these seven clinical specialists would not be interpreted to suggest the superiority of deep learning or machine learning algorithms to humans in identifying schizophrenia based on structural MRI data sets. Recent studies in other medical fields have compared humans and machine learning algorithms (50) and suggested that for the best performance of artificial intelligence, augmenting human intelligence is necessary (51).

There are several limitations to this study. All MRI data used in training the deep learning algorithm had binary labels (schizophrenia or normal). This dichotomous classification is widely used in studies of artificial intelligence, but it can be a barrier to applying this system in clinical practice. Most psychiatric diseases develop over a continuous spectrum (52), and multiple illnesses can coexist in a patient. As our analysis did not include a clinical comparison group, further study including other psychiatric illnesses, such as bipolar spectrum and neurodegenerative disorders, is needed. Because of this lack of clinical control groups, it is difficult to infer that the observed features of schizophrenia (i.e., medial temporal lobe abnormalities) are distinctive to schizophrenia.

Furthermore, within the data sets, there was no specific information regarding details of the illness (e.g., the presence of positive and negative symptoms of schizophrenia or the number of episodes). Thus, it was unclear whether the trained deep learning algorithm could discriminate progressive morphological changes in the patients with schizophrenia from the features of healthy controls.

Another crucial limitation is whether schizophrenia can be diagnosed solely by structural features of the brain. Unlike other diseases that can be accurately detected by photographs (13), schizophrenia is a disease accompanied by both structural and functional abnormalities. Recent studies have shown that functional MRI data and artificial intelligence techniques can also be used to reliably identify schizophrenia (53); thus, combining structural and functional features of brain images would be expected to increase the potential for clinical usage. Another limitation is that the region of interest was not specified in our regional analysis, in which one of eight regions in transverse sections was roughly occluded. A more detailed cortical parcellation might be required to accurately match the regions that contributed the most to deep learning with regions identified in previous studies using voxel-based analyses. The inconsistent data quality within the data set is another limitation. For example, the MCICShare and NUSDAST data sets had MRI data collected using a 1.5 T scanner, which has a lower signal-to-noise ratio and image resolution than data collected using a 3 T scanner. This low-image-quality data set could have obscured the performance of the algorithm. Finally, we should note that the results obtained from seven clinicians do not imply that the ability of humans to identify schizophrenia from MR images is decreased compared to that of deep learning algorithms. Cautious interpretation of the results is needed because the clinical specialists in this study were not experts in diagnosing psychiatric illnesses through MR images.

## Conclusions

Deep neural networks trained with multicenter structural MRI data sets showed high sensitivity and specificity in identifying schizophrenia. The developed deep learning algorithms identified schizophrenia fairly well in a new MRI data set acquired by a single center in which the disease characteristics of the patients were somewhat different. The deep learning algorithm depended mainly on information from the right temporal area in classifying schizophrenia. Deep learning algorithms trained with large data sets consisting of various stages and severities of illnesses could help clinicians discriminate schizophrenia from other psychiatric diseases and delineate the particular structural and functional characteristics of the brain in patients with schizophrenia.

## Data Availability Statement

The datasets generated for this study are available on request to the corresponding authors.

## Ethics Statement

The studies involving human participants were reviewed and approved by Institutional Review Board of Seoul St. Mary’s Hospital (KC18ZESI0615). Written informed consent for participation was not required for this study in accordance with the national legislation and the institutional requirements.

## Author Contributions

Concept and design: JO, KY. Data acquisition: JO, K-UL. Analysis: JO, KY. Data interpretation: KY, JO, B-LO, K-UL. Drafting of the manuscript: JO, KY. Critical revision of the manuscript: J-HC, B-LO, K-UL. Obtaining funding: JO.

## Funding

This study was supported by a grant of the Korea Health Technology R&D Project through the Korea Health Industry Development Institute (KHIDI), funded by the Ministry of Health & Welfare, Republic of Korea (grant number: HL19C0007) and the Research Fund of Seoul St. Mary’s Hospital, The Catholic University of Korea.

## Conflict of Interest

The authors declare that the research was conducted in the absence of any commercial or financial relationships that could be construed as a potential conflict of interest.

## Supplementary Material

Supplementary Figure 1 A Sample Process of Converting 3D MR Images to Video Format.

Supplementary Figure 2 A full model of a deep learning algorithm.

Supplementary Figure 3 Learning effect of psychiatrists.

Supplementary Figure 4 Training curve of the algorithm.

Supplementary Table 1 Scan Parameters of 5 Public Data Sets and Validation Data Set.

Supplementary Table 2 Confusion Matrix of Individual Clinicians in Detecting Schizophrenia through MR Images.

Supplementary Video 1 A Sample Video of Converted MR images.

Supplementary Video 2 A Sample Video of Converted MR images with occulded section.
