# Supplementary material for: Identifying Schizophrenia Using Structural MRI With a Deep Learning Algorithm
Source: Front Psychiatry. 2020 Feb 3;11:16. doi: 10.3389/fpsyt.2020.00016 (PMC7008229; doi:10.3389/fpsyt.2020.00016)

Supplementary Figure 1. A Sample Process of Converting 3D MR Images to Video Format

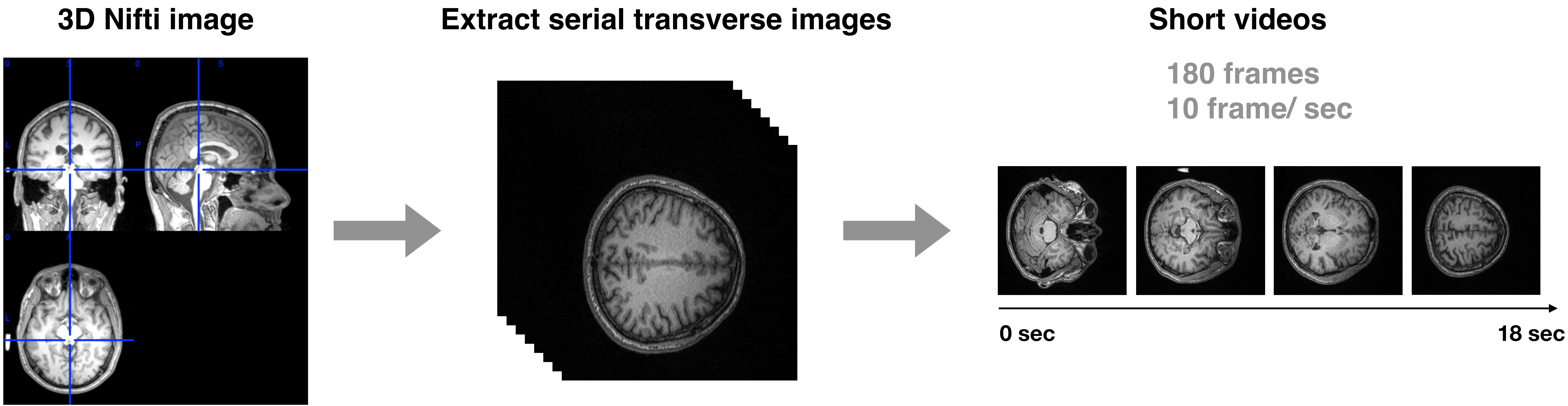

Supplement: Supplementary Figure 1 — A Sample Process of Converting 3D MR Images to Video Format. [file Image_1.pdf]
