# Supplementary figures and images for: Identifying Schizophrenia Using Structural MRI With a Deep Learning Algorithm
Source: Front Psychiatry. 2020 Feb 3;11:16. doi: 10.3389/fpsyt.2020.00016 (PMC7008229; doi:10.3389/fpsyt.2020.00016)

# Supplementary Figure 2

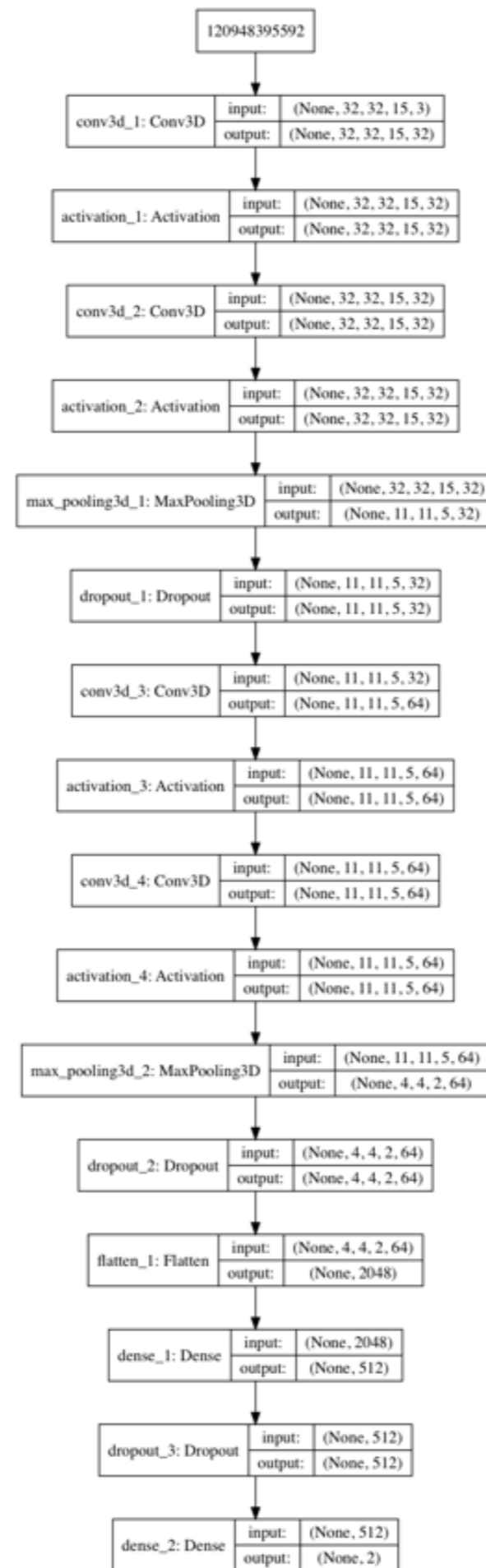

Supplement: Supplementary Figure 2 — A full model of a deep learning algorithm. [file Image_2.pdf]

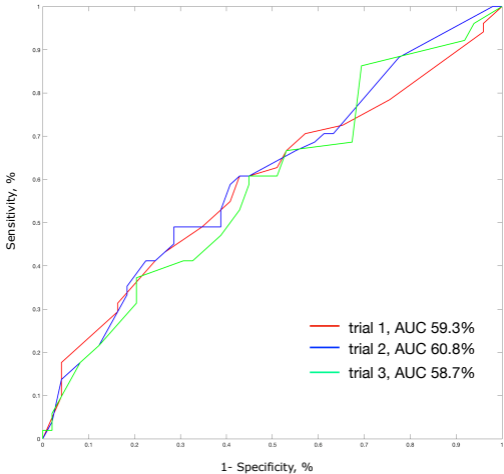

Supplement: Supplementary Figure 3 — Learning effect of psychiatrists. [file Image_3.pdf]

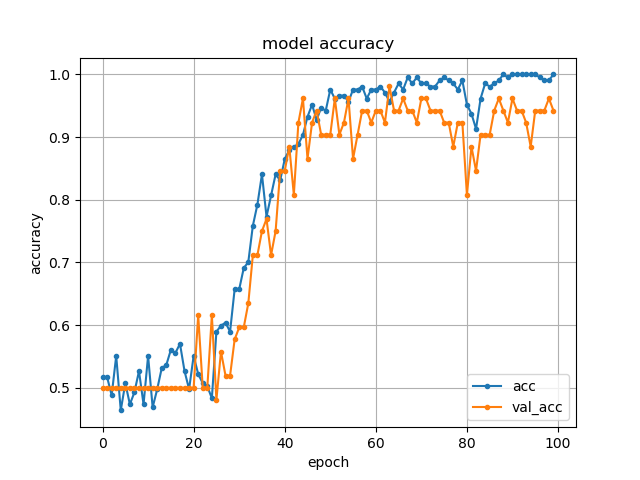

Supplement: Supplementary Figure 4 — Training curve of the algorithm. [file Image_4.png]
