# Supplementary material for: Identifying Schizophrenia Using Structural MRI With a Deep Learning Algorithm
Source: Front Psychiatry. 2020 Feb 3;11:16. doi: 10.3389/fpsyt.2020.00016 (PMC7008229; doi:10.3389/fpsyt.2020.00016)
Supplement: Supplementary Table 1 — Scan Parameters of 5 Public Data Sets and Validation Data Set. [file Table_1.pdf]

Supplementary Table 1. Scan Parameters of 5 Public Data Sets and Validation Data Set

Scan Parameters	BrainGluSchi[a]	COBRE[b]	MCICShare (3T/1.5T)[c]	Nmorph[d]	NUSDAST[e]	Ujeongbu St. Mary's
Scanner	SIEMENS TrioTim syngo MR B17	SIEMENS Triotim syngo MR B17	SIEMENS Trio/ SIEMENTS Sonata	-	-	SIEMENS Avanto
Sequence	MPRAGE	MPRAGE	MPRAGE	MPRAGE	MPRAGE	MPRAGE
Voxel size (mm)	1.0x1.0x1.0	1.0x1.0x1.0	0.625x0.625x1.5	1.0x1.0x1.6	1.0x1.0x1.25	1.0x1.0x1.0
Matrix size	256x256	256x256	256x256	256x256	256x256	256x256
TR (msec)	2530	2530	2530/12	3.15	9.7	1090
TE (msec)	1.64	1.64	3.79/4.76	20	4	4.24
Flip angle (deg)	7.0	7.0	7/20	8.0	10	9
Signal to Noise Ratio	1.00	1.00	-	-	-	-

[a] http://schizconnect.org/uploads/data_instruction/pdffile/7/Braingluschi_Scan_Parameters.pdf

[b] http://schizconnect.org/uploads/data_instruction/pdffile/2/COBRE_Scan_Information.pdf

[c] Gollub, R.L., Shoemaker, J.M., King, M.D et al., (2013). The MCIC collection: a shared repository of multi-modal, multi-site brain image data from a clinical investigation of schizophrenia. *Neuroinformatics*, *11*(3), 367-388.

[d] http://schizconnect.org/uploads/data_instruction/pdffile/5/NMorphCH_data_description.pdf

[e] http://schizconnect.org/uploads/data_instruction/pdffile/1/NUSDAST_Data_150511.pdf.
