# Supplementary material for: Identifying Schizophrenia Using Structural MRI With a Deep Learning Algorithm
Source: Front Psychiatry. 2020 Feb 3;11:16. doi: 10.3389/fpsyt.2020.00016 (PMC7008229; doi:10.3389/fpsyt.2020.00016)
Supplement: Supplementary Table 2 — Confusion Matrix of Individual Clinicians in Detecting Schizophrenia through MR Images. [file Table_2.pdf]

Supplementary Table 2. Confusion Matrix of Individual Clinicians in Detecting Schizophrenia through MR Images

	Accuracy rate (%)	True Positive Rate (%)	False Positive Rate (%)	True Negative Rate (%)	False Negative Rate (%)
**Radiologist 1**	60	62.2	37.8	58.2	41.8
**Radiologist 2**	56	57.8	42.2	54.5	45.5
**Psychiatrist 1**	59	64.7	35.3	56.1	43.9
**Psychiatrist 2**	53	54.5	45.5	51.8	48.2
**Psychiatrist 3**	48	51.1	48.9	49.1	50.9
**Psychiatrist 4**	58	69.6	30.4	54.5	45.5
**Psychiatrist 5**	45	45.7	54.3	44.4	55.6
